# Exploring the molecular mechanisms of Pueraria in Alzheimer’s disease treatment using machine learning and network pharmacology

**DOI:** 10.3389/fnut.2025.1683852

**Published:** 2025-10-21

**Authors:** Kai Ye, Li Li, Li Guan, Ming-ming Qin, Xue-ying Xu, Jing Wu, Li-zhu Huang, Jun-jie Gao

**Affiliations:** ^1^Department of Clinical Laboratory, The Second Affiliated Hospital of Wannan Medical College, Wuhu, China; ^2^Department of Clinical Laboratory, The First Affiliated Hospital of Wannan Medical College (Yijishan Hospital of Wannan Medical College), Wuhu, China; ^3^Department of Hepatology, Wuhu Third People’s Hospital, Wuhu, China

**Keywords:** Pueraria, Alzheimer’s disease, network pharmacology, machine learning, neuroprotection

## Abstract

**Background:**

Alzheimer’s disease (AD) is a multifactorial neurodegenerative disorder, characterized by amyloid-*β* deposition, tau pathology, neuroinflammation, and metabolic dysfunction. While conventional treatments have been widely studied, food-based interventions are emerging as potential neuroprotective strategies. Pueraria, a nutrient-rich food, has shown promise in promoting brain health, but its mechanisms in AD prevention and management remain insufficiently understood.

**Methods:**

In this study, we utilized network pharmacology, transcriptomics, and machine learning to investigate the neuroprotective effects of Pueraria. Through analysis of five transcriptomic datasets (GSE5281, GSE29378, GSE36980, GSE37263, and GSE138260), we identified genes associated with AD and screened 15 active compounds from *Pueraria lobata* using HERB and TCMSP databases. Machine learning models prioritized key targets, and molecular docking simulations assessed the binding affinities of Pueraria compounds to these targets. *In vivo* validation was performed in AD model mice to evaluate the cognitive-enhancing effects of Pueraria.

**Results:**

We identified 45 overlapping targets between Pueraria and AD, primarily related to synaptic plasticity and neurotransmission. Among these, PFKFB3 emerged as a key mediator of Pueraria’s neuroprotective effects. Molecular docking confirmed strong binding affinities between Pueraria compounds and PFKFB3, supporting their functional role. Experimental data showed that Pueraria improved cognitive function in AD mice, underscoring its potential as a neuroprotective agent.

**Conclusion:**

This study highlights Pueraria as a promising functional food for AD prevention and management, emphasizing the potential of plant-based dietary interventions for brain health. Our findings provide a basis for further exploration of food-derived neuroprotective strategies.

## 1 Introduction

Alzheimer’s disease (AD) is the most prevalent neurodegenerative disorder in the elderly, characterized by progressive cognitive decline and memory loss (1). As the global population ages, the prevalence of AD is rising, presenting a significant public health challenge. Projections suggest that by 2050, more than 139 million people will be living with AD and related dementias (2, 3). Underscoring the urgent need for effective prevention and intervention strategies. Despite decades of research, AD remains incurable, and current treatments fail to substantially slow disease progression (4, 5). The pathogenesis of AD is multifactorial, involving intricate interactions between genetic, environmental, and lifestyle factors (6, 7). Key pathological features of AD include the accumulation of amyloid-*β* plaques, tau tangles, neuroinflammation, and mitochondrial dysfunction. These processes collectively contribute to synaptic dysfunction, neuronal loss, and cognitive decline (8–10). Traditional drug therapies typically target individual factors, such as amyloid-*β* or tau, but their limited efficacy highlights the need for more comprehensive approaches. Consequently, there has been growing interest in holistic, food-based interventions capable of addressing multiple pathways in AD pathology simultaneously (11, 12).

In this context, functional foods are emerging as promising candidates for the prevention and management of chronic diseases like AD (13, 14). Pueraria, a plant native to East Asia, has garnered attention for its diverse array of bioactive compounds, many of which exhibit neuroprotective properties (15, 16). Widely used in East Asian culinary practices, Pueraria is incorporated into soups, teas, and starches, enhancing flavor and texture while potentially offering health benefits (17, 18). Active compounds in Pueraria, including daidzein, puerarin, and genistein, have been shown to modulate key AD-related pathways (19, 20). These compounds reduce amyloid-*β* production, enhance its clearance, inhibit tau hyperphosphorylation, and protect against neuroinflammation and oxidative stress (21, 22). Although most research has focused on the medicinal benefits of Pueraria, its potential as a dietary supplement remains underexplored. Given the growing interest in functional foods and the increasing demand for natural, plant-based alternatives to synthetic drugs, exploring the neuroprotective effects of Pueraria as part of a daily diet is essential.

**Figure 1:**
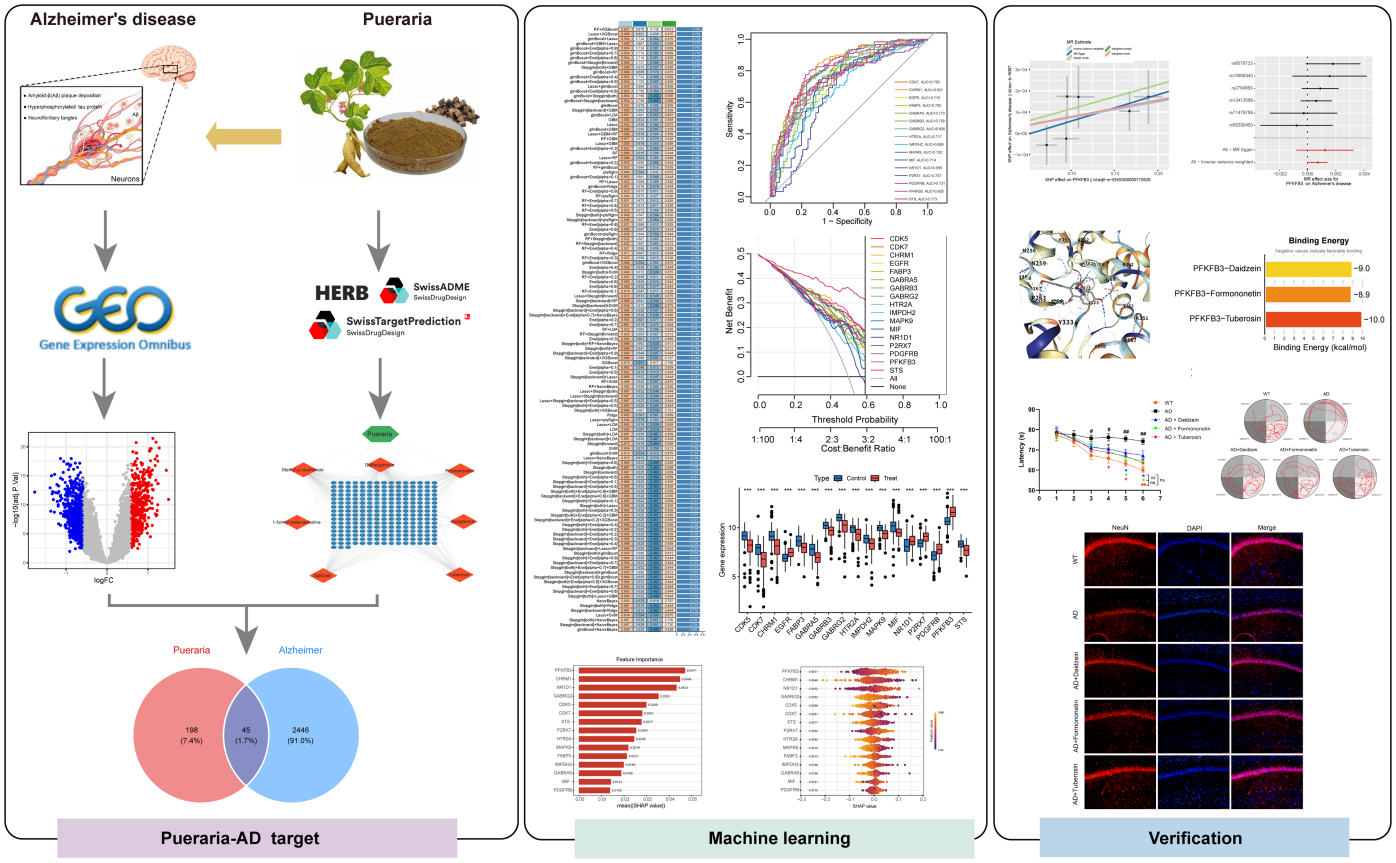
Complete analysis workflow.

This study aims to investigate the neuroprotective mechanisms of Pueraria from a food perspective, using network pharmacology, transcriptomics, and machine learning to understand how its bioactive compounds interact with key molecular targets in AD. By evaluating Pueraria as a functional food, we highlight its potential role in AD prevention and management and offer new insights into plant-based neuroprotective strategies. Figure 1 shows the study flow chart.

## 2 Materials and methods

### 2.1 Animal experiments

Male C57BL/6J mice were purchased from Jiangsu Jicui Yao Kang Biotechnology Co., Ltd. All animals were housed in the Animal Experiment Center of the Experimental Center of Wannan Medical College under standard laboratory conditions, and all procedures were conducted in accordance with relevant animal welfare guidelines. All experimenters were certified in experimental animal handling, and the protocol was approved by the Animal Ethics Committee of Wannan Medical College (Approval Number: WNMC-AWE-2025199). At 16 weeks of age, mice underwent behavioral assessment of learning and memory functions using the Morris water maze (MWM), novel object recognition (NOR), and Y-maze tests. All behavioral testing was performed during the animals’ active photoperiod (08:00–12:00) with *n* = 8 mice per group for each behavioral test (MWM, NOR, and Y-maze).

### 2.2 Construction of the Abeta_1-42_-induced Alzheimer’s disease mouse model

Aβ_1-42_ was dissolved in 1% NH₃·H₂O to a concentration of 1 μg/μL and incubated at 37 °C for 5 days to promote oligomer (fibril) formation. Following skull drilling at the designated stereotactic coordinates using a dental drill, Aβ_1-42_ (4 μg) was bilaterally injected into the hippocampi of male C57BL/6 mice using a microinjector. After injection, the scalp was sutured, and mice were maintained at 37.5 °C ± 0.5 °C. Behavioral testing commenced 2 weeks after injection.

### 2.3 Morris water maze test

The MWM test was used to evaluate spatial learning and long-term memory. A circular pool was divided into four quadrants, each marked by distinctive wall patterns and colors (red, green, yellow, blue) serving as spatial cues. The transparent circular platform was placed in the target quadrant, designated as the third quadrant in the software system, with the farthest quadrant designated as the first quadrant. Each trial lasted 60 s, and mice were required to remain on the platform for 5 s upon location.

### 2.4 Novel object recognition test

The NOR test was used to assess short-term working memory. Mice were habituated to a dimly lit plastic box (without objects) for 10 min. During the familiarization phase, two identical cube-shaped objects (A and B; 3 cm sides) were placed 8.5 cm from the left and right box walls in the front third of the arena. Mice were allowed to explore for 10 min, and exploration trajectories, time, and frequency were recorded. After a 1-h interval, the test phase was conducted by replacing object B with a novel cylindrical object (C) of similar volume but different color and shape, positioned identically. Mice were placed back in the same starting position, and exploration data were recorded.

### 2.5 Y-maze test

The Y-maze test assessed hippocampus-dependent spatial working memory. Mice were allowed to freely explore the three arms for 5 min, during which the sequence and frequency of arm entries were recorded. The time spent in the starting arm, central area, and novel arm was analyzed.

### 2.6 Immunofluorescence staining

After 21 days of continuous administration, eight mice per group were anesthetized and transcardially perfused with ice-cold 4% paraformaldehyde. Brains were removed, post-fixed overnight in 4% paraformaldehyde, and cryoprotected sequentially in 20 and 30% sucrose/PBS at 4 °C (overnight each). Tissues were embedded in optimum cutting temperature compound (Sakura Finetek, United States) and coronally sectioned at 30 μm thickness using a cryostat. Sections were blocked with 10% fetal bovine serum in PBS for 1 h at room temperature, incubated overnight at 4 °C with mouse anti-NeuN primary antibody (ab104224, Abcam), and then incubated for 30 min at 37 °C with Alexa Fluor–conjugated secondary antibody (Invitrogen). Nuclei were counterstained with 4′,6-diamidino-2-phenylindole (DAPI). Images were acquired using a Leica TCS SP8 confocal microscope. Throughout the 21-day experimental period, all mice were monitored daily for signs of toxicity, including body weight changes, behavioral abnormalities, food and water consumption, and general health status. Body weights were recorded every 3 days. No adverse effects, mortality, or significant body weight changes (>10% loss) were observed in any treatment group. Additionally, gross necropsy examination revealed no visible organ abnormalities in treated animals.

### 2.7 Transcriptome data processing

Five Alzheimer’s disease-related transcriptomic datasets (GSE5281, GSE29378, GSE36980, GSE37263, and GSE138260) were obtained from the Gene Expression Omnibus (GEO) database, comprising gene expression profiles from AD patients and healthy controls. Batch effects among datasets were corrected using the ComBat algorithm in the sva R package (v3.40.0, R v4.1.0). Principal component analysis (PCA) was performed to assess sample distribution before and after correction, confirming effective removal of technical variability. Differentially expressed genes (DEGs) were identified using the limma package (v3.48.3) with criteria |log₂FC| >0.585 (1.5-fold change) and adjusted *p* ≤ 0.05. The ggplot2 package was used to generate volcano plots for DEG distribution visualization, and the pheatmap package was used to construct heatmaps displaying DEG expression patterns between groups.

### 2.8 Weighted gene co-expression network analysis

Gene co-expression networks were constructed using the weighted gene co-expression network analysis (WGCNA) package (version 1.71) to identify disease-related gene modules. Pearson correlation coefficients were calculated for all gene pairs, and a soft threshold of *β* = 5 was selected to ensure scale-free topology (*R*^2^ > 0.80). The dynamic tree cutting algorithm was employed to cluster genes into modules, with a minimum module size of 60 genes. Module eigengenes (MEs) were computed for each module, and Pearson correlation analysis was performed to assess the association between modules and Alzheimer’s disease (AD) phenotype. Modules significantly associated with AD were selected and merged with differentially expressed genes (DEGs) identified through Venn diagram analysis, yielding candidate genes linked to AD.

### 2.9 Network pharmacology analysis and target prediction of Pueraria

Chinese medicine databases, including HERB and TCMSP, were searched to identify the main bioactive components of Pueraria, such as puerarin, daidzein, and genistein. Potential target proteins for these compounds were predicted using the Swiss Target Prediction platform, with a probability threshold set at >0.5 for reliable target identification. Cytoscape software (version 3.9.1) was utilized to visualize the interactions between Pueraria bioactive components and their target proteins, constructing a network of multi-component and multi-target interactions.

### 2.10 Enrichment analysis

Common target genes, identified through Venn diagram analysis of the intersection between predicted Pueraria targets and AD-associated genes, were subjected to Gene Ontology (GO) functional annotation and Kyoto Encyclopedia of Genes and Genomes (KEGG) pathway enrichment analysis using the cluster Profiler package (version 4.0.5). The Benjamini–Hochberg method was used to adjust *p*-values, and a corrected *p*-value <0.05 was considered statistically significant.

### 2.11 Machine learning model construction and core gene selection

The merged transcriptomic datasets (GSE5281 and GSE36980) were used for model training, while three independent datasets (GSE138260, GSE29378, and GSE37263) served as external validation sets. Machine learning models were constructed from 127 different combinations, incorporating classification algorithms (Random Forest, Support Vector Machine, XGBoost, Logistic Regression, and Neural Networks) and feature selection methods (LASSO regression, recursive feature elimination, and ANOVA). Ensemble learning strategies, such as voting and stacking, were employed to improve model performance. The models were evaluated via 10-fold cross-validation, using metrics including the area under the receiver operating characteristic curve (AUC), sensitivity, specificity, accuracy, and F1 score. The RF + XGBoost ensemble model (average AUC = 0.792) was selected for further analysis due to its optimal performance in both the training and validation sets. Based on feature importance rankings, 17 core genes with AUC values exceeding 0.6 in individual ROC analyses were identified. Decision curve analysis (DCA) was applied to assess the clinical net benefit of these core genes at various threshold probabilities.

### 2.12 SHAP interpretability analysis

The SHAP framework (version 0.41.0) was employed to quantify the contribution of the 17 core genes to the machine learning model’s predictions. SHAP values and average absolute SHAP values were calculated for each gene, and importance ranking and swarm plots were generated to illustrate the direction and strength of each gene’s impact on AD prediction.

### 2.13 Mendelian randomization analysis

A two-sample Mendelian randomization (MR) approach was applied to assess the causal association between core genes and AD. Genetic variants linked to PFKFB3 and NR1D1 gene expression were sourced from the GWAS database as instrumental variables (eQTL *p*-value <5 × 10^−8^). Genetic association data for AD were obtained from the GWAS database (ieu-b-5067). Causal effects were estimated using inverse variance weighting, MR-Egger regression, and weighted median methods. The Cochran *Q* test was applied to evaluate heterogeneity, while the MR-PRESSO method detected horizontal pleiotropy. The leave-one-out approach was used to validate the robustness of the results.

### 2.14 Molecular docking validation

The three-dimensional crystal structure of PFKFB3 was retrieved from the Protein Data Bank (PDB) and preprocessed using PyMOL software (version 2.5.2), removing water and other ligands, adding polar hydrogens, and assigning Gasteiger charges with AutoDock Tools. The chemical structures of key Pueraria active compounds (daidzein, formononetin, and tuberosin) were obtained from PubChem and optimized using Avogadro software (version 1.95.0) with the MMFF94 force field. Molecular docking simulations were conducted using AutoDock Vina software (version 1.2.0), with the active site of PFKFB3 defined as a 10 Å radius from the natural ligand. Docking results were evaluated based on binding energy (kcal/mol), with binding energies ≤−5.0 kcal/mol indicating strong interactions. The best docking conformations were visualized using PyMOL software to analyze protein-ligand interactions and key amino acid residues involved in binding.

### 2.15 Statistical analysis

All statistical analyses were performed using R software. Continuous variables were compared using Student’s *t*-test or Mann–Whitney *U* test, while categorical variables were assessed using the chi-square test. Multiple comparison corrections were performed using the Benjamini–Hochberg method to control the false discovery rate. A two-sided *p*-value <0.05 was considered statistically significant. Mendelian randomization analyses were conducted using the TwoSampleMR package, and molecular docking results were validated through multiple independent runs to ensure reproducibility. Quantitative data are presented as mean ± standard deviation.

## 3 Result

### 3.1 Screening of differentially expressed genes in Alzheimer’s disease and weighted gene co-expression network analysis

**Figure 2:**
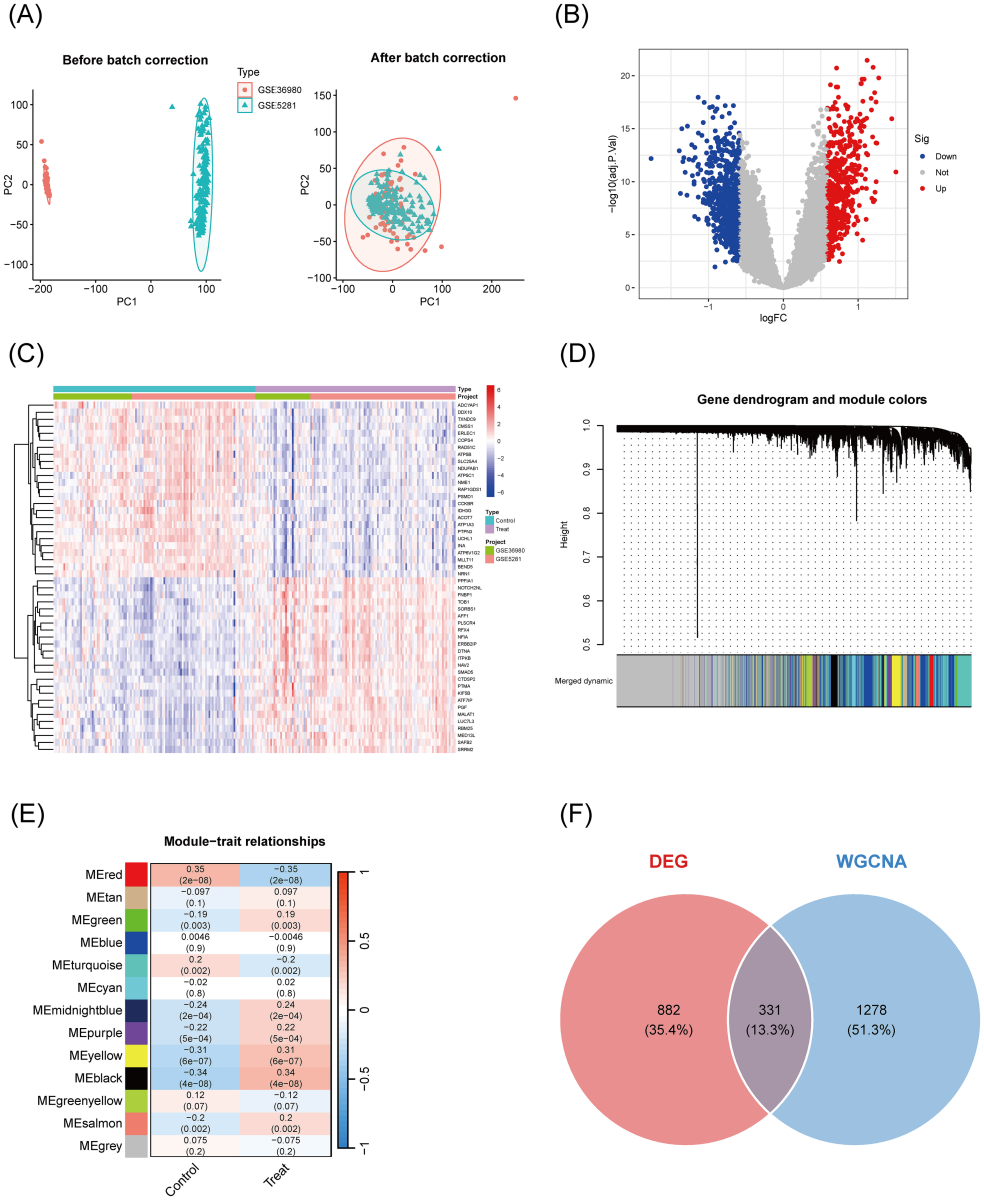
Visualization of differentially expressed genes in Alzheimer’s disease transcriptomic data and WGCNA feature genes. **(A)** PCA plots before and after batch correction: The horizontal axis (PC1) and vertical axis (PC2) show the distribution of samples; the left plot shows the distribution before correction (red circles for GSE36980, blue triangles for GSE5281), and the right plot shows the distribution after correction, with mixed samples and ellipses indicating overlapping distributions, confirming that batch effects have been removed. **(B)** Volcano plot of differentially expressed genes: Displays the significance of gene expression changes, with the horizontal axis representing logFC (from −1 to 1) and the vertical axis representing −log10(adj. *p*-value) (from 0 to 20). Red points indicate significantly upregulated genes, blue points indicate significantly downregulated genes, and gray points represent non-significant genes. **(C)** Heatmap of differentially expressed genes: Visualizes the expression patterns of 50 DEGs in the control group (left) and disease group (right). Red indicates high expression (logFC >0), blue indicates low expression (logFC <0), and white indicates intermediate levels, showing significant expression differences between groups. **(D)** Gene clustering tree and module color map: Genes are shown on the *x*-axis, and clustering height is on the *y*-axis. The color bars below indicate different modules (e.g., “merged dynamic”) used for gene module partitioning. **(E)** Module-trait relationship heatmap: Modules are on the *x*-axis, and correlations are on the *y*-axis. The color intensity (red = positive correlation, blue = negative correlation) and numerical values (correlation coefficients and *p*-values) indicate the strength of the association between modules and traits. **(F)** Venn diagram: The orange circle represents DEGs, and the blue circle represents WGCNA feature genes. The numbers indicate the size of the intersection, identifying key candidate genes.

To investigate the gene expression patterns associated with Alzheimer’s disease (AD) and integrate multi-dataset analysis, batch correction was first performed on the gene expression data from the GSE36980 and GSE5281 datasets. Principal component analysis (PCA) confirmed that the batch effect was successfully removed, with the corrected samples uniformly distributed (Figure 2A), indicating effective batch correction. Differentially expressed genes (DEGs) were identified using a volcano plot, which revealed 558 significantly upregulated genes (logFC >0.585 and adj. *p*-value ≤0.05) and 655 significantly downregulated genes (logFC <−0.585 and adj. *p*-value ≤0.05) (Figure 2B). A heatmap of 25 DEGs showed clear patterns of high expression (red) and low expression (blue) in the disease group compared to the control group (Figure 2C). Additionally, weighted gene co-expression network analysis (WGCNA) was applied to construct a gene clustering tree and partition genes into modules (Figure 2D). Module-trait relationship analysis revealed three modules—MEred, MEyellow, and MEblack—that were highly correlated with disease traits (Figure 2E). Finally, a Venn diagram was used to merge the DEGs identified for Alzheimer’s disease with the characteristic genes from the three WGCNA modules, yielding a total of 2,491 potential target genes for AD (Figure 2F).

### 3.2 Identification of common targets between Pueraria and Alzheimer’s disease and functional enrichment analysis

**Figure 3:**
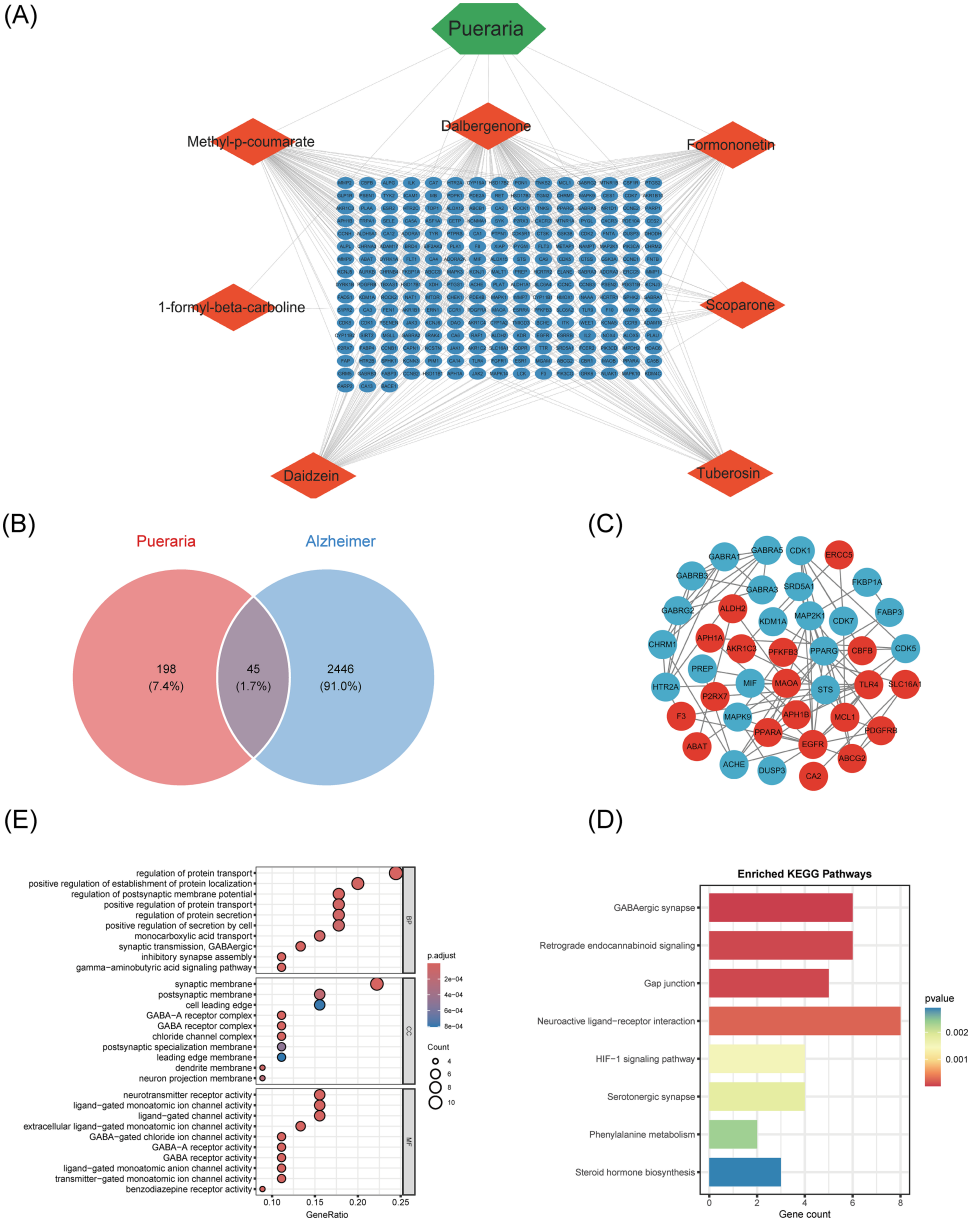
Identification of common targets between Pueraria and Alzheimer’s disease and functional enrichment analysis. **(A)** Interaction network between the active components of Pueraria (diamond nodes) and target genes (hexagonal nodes, 236 in total), showing multi-component-multi-target regulatory characteristics through the connections. **(B)** Venn diagram showing the intersection of 45 genes (1.7%) between Pueraria target genes (orange circle, 198 genes, 7.4%) and AD characteristic genes (blue circle, 2,446 genes, 91.0%), indicating potential intervention targets. **(C)** Expression pattern network diagram of the intersecting genes in AD, with red nodes (increased expression) and blue nodes (decreased expression), and connections representing gene interaction relationships. **(D)** GO enrichment analysis of the intersection genes, presented in bar (left) and bubble (right) charts. The analysis shows significant enrichment in cellular components, biological processes, and molecular functions, with gene ratio and adjusted *p*-values. **(E)** Bar chart of KEGG pathway enrichment for the intersecting genes, with the vertical axis representing pathways and the horizontal axis representing the number of genes. The color gradient indicates enrichment significance, with red indicating smaller *p*-values.

To explore the molecular mechanisms underlying the therapeutic effects of Pueraria on Alzheimer’s disease (AD), we integrated network pharmacology and bioinformatics approaches. First, an interaction network was constructed between the active components of Pueraria and 236 target genes (Figure 3A). A Venn diagram was used to identify the intersection between Pueraria target genes and AD-associated genes, revealing 45 common genes (Figure 3B). We then analyzed the expression changes of these 45 genes in AD (Figure 3C) and performed functional annotation. GO enrichment analysis highlighted their significant involvement in synaptic plasticity, GABA-A receptor activity, and other neural functions (Figure 3D). KEGG pathway analysis revealed that these genes are associated with core pathways, including GABAergic synapses and retrograde endocannabinoid signaling (Figure 3E). These findings suggest that Pueraria regulates AD-related neural pathways through these 45 key target genes, supporting its multi-component, multi-target action as a therapeutic agent for AD.

### 3.3 Core therapeutic target screening and validation based on machine learning algorithms

**Figure 4:**
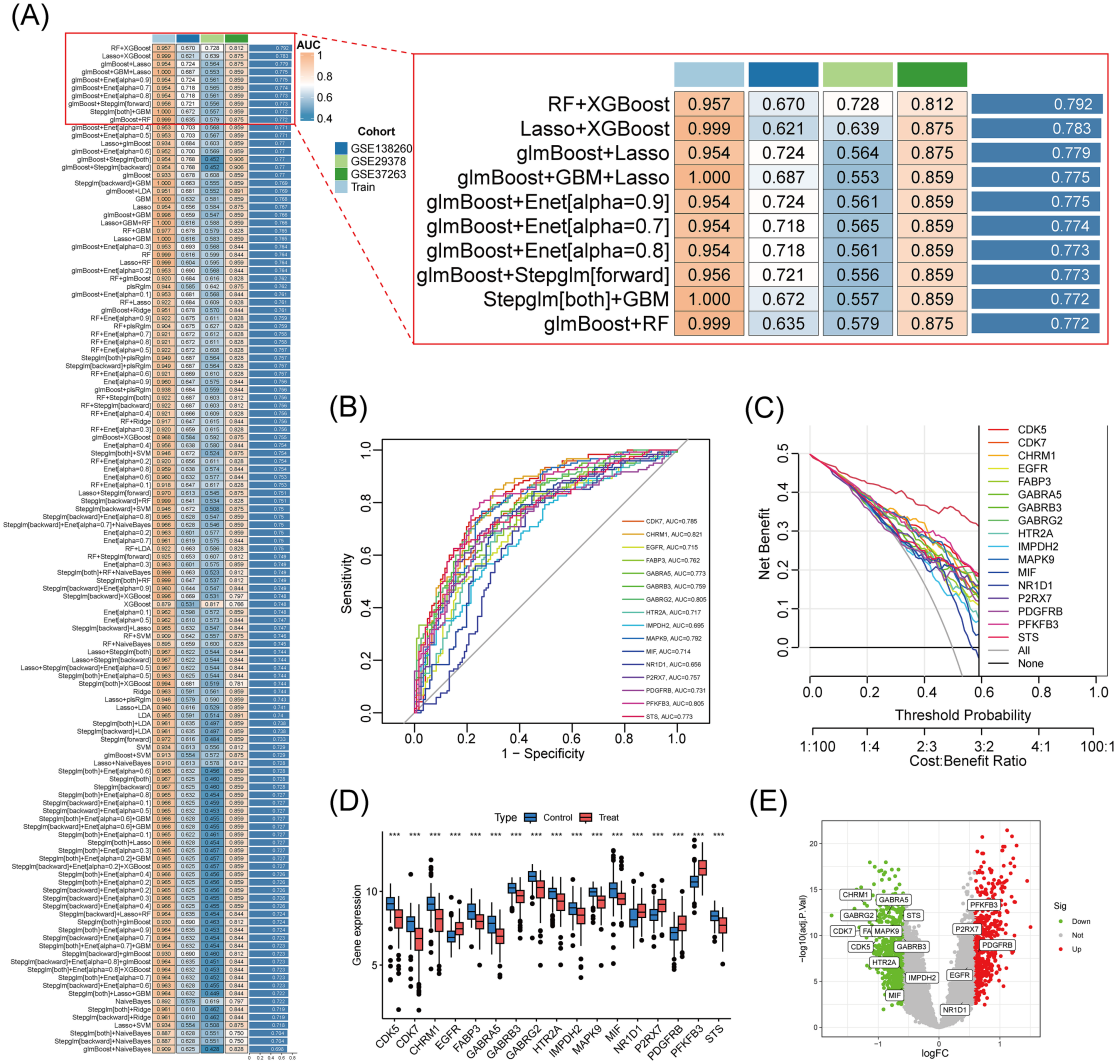
Core therapeutic target screening and validation based on machine learning algorithms. **(A)** Heatmap of the results from 127 machine learning models: Shows the AUC values of different model combinations on the training and test sets. Color gradients represent performance levels (blue for low values and red for high values). **(B)** ROC curves for 17 core genes: The *x*-axis represents specificity, and the *y*-axis represents sensitivity. The curves display the classification performance of each gene, with AUC values ranging from 0.715 to 0.829. **(C)** Decision curves for 17 core genes: The horizontal axis represents the threshold probability, and the vertical axis represents net benefit. The curves assess clinical utility at different cost-effectiveness ratios, with higher curves indicating greater net benefits. **(D)** Box plot of the expression of 17 core genes in Alzheimer’s disease: Compares gene expression levels between AD patients (Treat) and control subjects (Control). **(E)** Volcano plot of the expression of 17 core genes: The *x*-axis represents logFC (threshold ±0.585 corresponds to a 1.5-fold difference), and the vertical axis represents −log10(adj. *p*-value) (threshold 1.3 corresponds to adj. *p*-value = 0.05). Red points indicate significantly upregulated genes, blue points indicate downregulated genes, and gray points indicate non-significant genes.

To assess the diagnostic value of the 45 intersecting genes from Pueraria for Alzheimer’s disease (AD), we analyzed the expression data of these genes using 127 machine learning models. The performance of each model was evaluated based on the area under the receiver operating characteristic curve (AUC) for both the training set and multiple independent test sets (GSE138260, GSE29378, GSE37263). The results indicated that the RF + XGBoost method performed optimally, with an average AUC of 0.792 on both the training and validation sets (Figure 4A). Using this RF + XGBoost algorithm, we identified 17 core genes associated with the therapeutic effects of Pueraria on AD. Receiver Operating Characteristic (ROC) analysis of these 17 core genes revealed AUC values greater than 0.6, with PFKFB3 achieving the highest AUC of 0.805 (Figure 4B). Decision curve analysis demonstrated that these genes provided a net benefit within threshold probabilities ranging from 0.2 to 0.8, with the maximum net benefit observed at approximately 0.4 (Figure 4C). Box plots (Figure 4D) and volcano plots (Figure 4E) illustrated the expression levels of these 17 core genes in the AD group. Among them, PFKFB3, P2RX7, PDGFRB, EGFR, and NR1D1 were significantly upregulated, while CHRM1, CDK5, CDK7, FABP3, GABRA5, GABRB3, GABRG2, HTR2A, IMPDH2, MAPK9, MIF and STS were significantly downregulated (*p* < 0.001).

### 3.4 Core target importance assessment and causal association validation analysis

**Figure 5:**
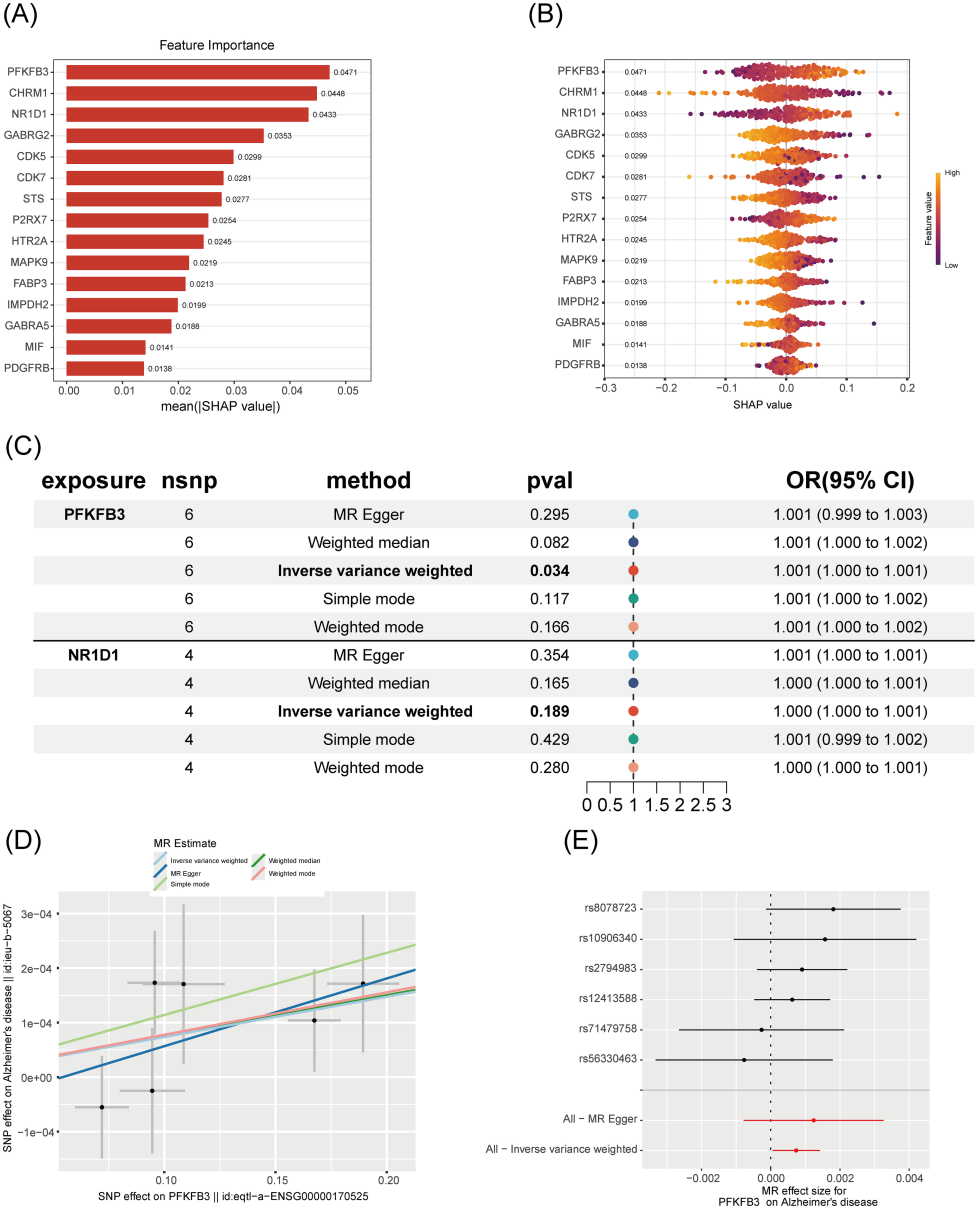
Comprehensive analysis of the importance and causal relationship of core genes in Alzheimer’s disease. **(A)** SHAP importance bar chart: Shows the average absolute SHAP value (mean (|SHAP value|)) for each gene, with higher values indicating greater importance in the machine learning model. Genes are sorted in descending order of importance. **(B)** SHAP swarm plot: Displays the distribution of SHAP values for each gene. Points represent individual samples, with colors indicating the magnitude of feature values (blue for low, red for high), visualizing the influence of gene feature values on prediction outcomes. **(C)** Mendelian randomization forest plot: Shows the odds ratio (OR) and 95% confidence interval for the association between genes and Alzheimer’s disease, using different MR methods. *p*-values are annotated to assess the significance of causal associations. **(D)** Mendelian randomization scatter plot: Illustrates the relationship between the effect of SNPs on PFKFB3 and their effect on Alzheimer’s disease, with colored points representing different estimation methods. This plot visually displays the direction of causal effects. **(E)** Mendelian randomization forest plot (SNP level): Shows the effect sizes and confidence intervals of individual SNPs and their combined methods, assessing the robustness of the causal association between PFKFB3 and Alzheimer’s disease.

To investigate the role and predictive value of core genes in Alzheimer’s disease (AD), we used the SHAP explainable machine learning method to assess gene importance and performed a Mendelian randomization (MR) experiment to evaluate causal associations. In the SHAP analysis, the gene PFKFB3 exhibited the highest importance, with a mean (|SHAP value|) of approximately 0.0471 (Figure 5A). The SHAP swarm plot further revealed that higher feature values of PFKFB3 (represented by red dots) positively impacted prediction, with SHAP values ranging from 0 to 0.2 (Figure 5B). In the Mendelian randomization analysis, the inverse variance-weighted method produced a *p*-value of 0.034 and an odds ratio (OR) of 1.001 (95% CI, 1.000 to 1.001), indicating a significant causal association between PFKFB3 and AD (Figure 5C). Scatter plots and forest plots further supported a positive causal relationship between PFKFB3 and Alzheimer’s disease (Figures 5D,E).

### 3.5 Molecular docking validation of active components of Pueraria and PFKFB3 target

**Figure 6:**
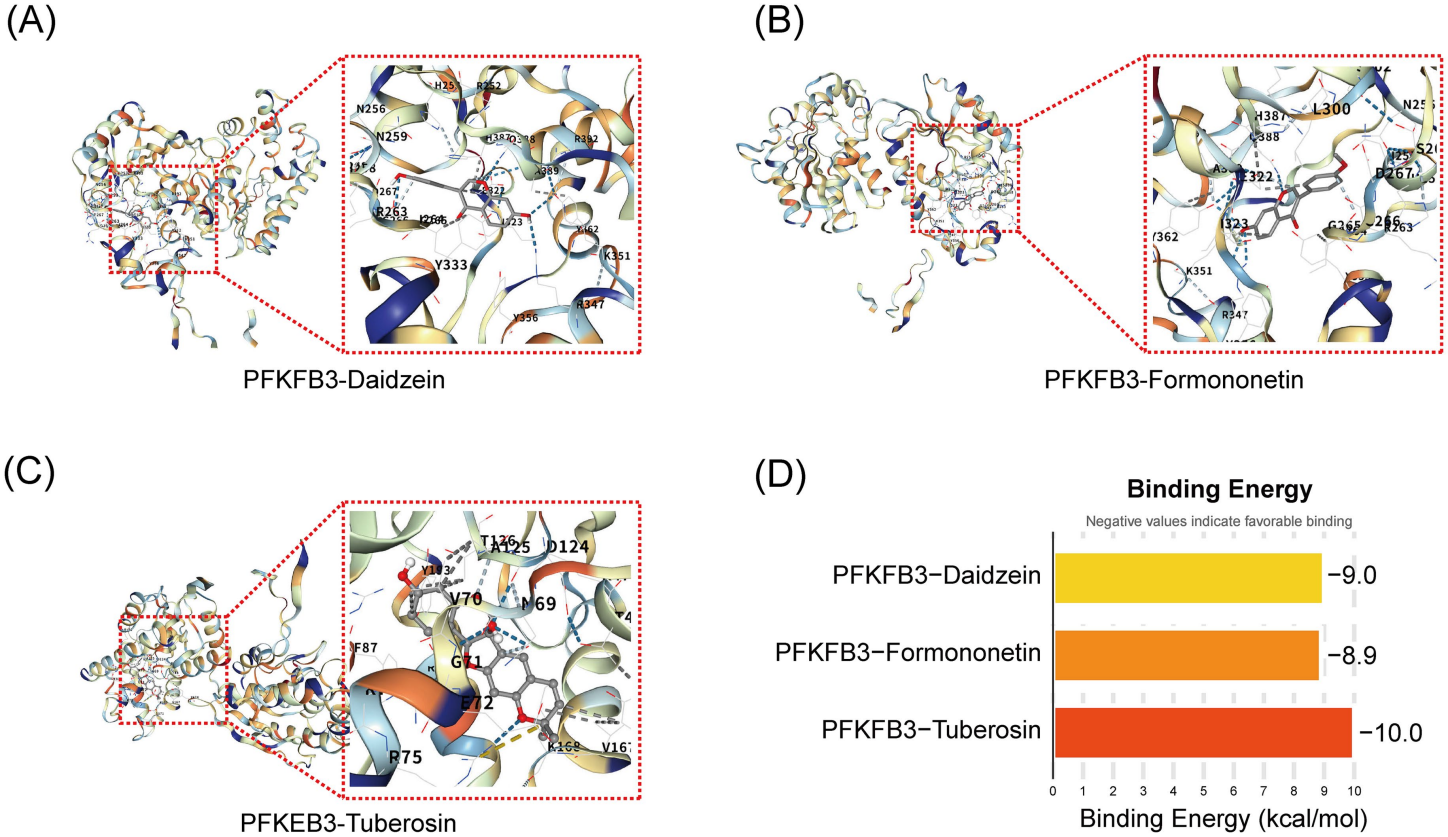
Molecular docking validation of active components of Pueraria and PFKFB3 target. **(A–C)** Binding conformation diagrams for the docking of daidzein, formononetin, and tuberosin with the PFKFB3 protein. **(D)** Target protein binding energy bar chart: The vertical axis represents different target proteins and active components (PFKFB3-daidzein, PFKFB3-formononetin, PFKFB3-tuberosin), while the horizontal axis represents binding energy (kcal/mol, range from −10 to 0). Column colors indicate binding energy values (light yellow to red), with lower values (more negative) representing stronger binding affinity.

To explore the potential binding interactions between PFKFB3 and the active components of Pueraria (daidzein, formononetin, and tuberosin), we performed molecular docking analysis. The results showed that PFKFB3 exhibited significant binding affinity with all three active components, with calculated binding energies consistently below −8.0 kcal/mol (Figure 6D). According to established criteria in molecular docking, a binding energy of <−5.0 kcal/mol indicates a strong binding affinity. Visualization of the primary binding conformations (Figures 6A–C) revealed stable docking complexes for each active component–protein pair. These findings provide structural evidence for direct molecular interactions between Pueraria and the core target PFKFB3.

### 3.6 Active components of Pueraria improve pathological behavior and neuronal loss in AD mice

**Figure 7:**
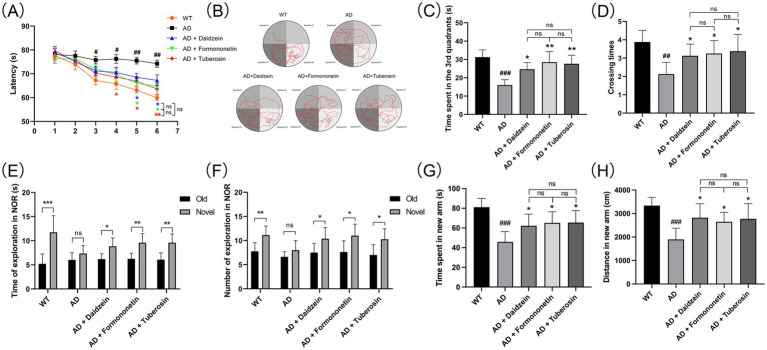
Pathological behavior of AD mice improved by active components of Pueraria. **(A–D)** MWM test results: Time to reach the platform **(A)**, escape trajectory **(B)**, time spent in the target quadrant **(C)**, and number of platform crossings **(D)**. **(E,F)** NOR test results: Time spent **(E)** and number of visits **(F)** to the novel object. **(G,H)** Y-maze test results: Time **(G)** and distance **(H)** spent in the new arm. All values are presented as mean ± SEM. ^##^*p* < 0.01 and ^###^*p* < 0.001 compared with the WT group by *t*-test. ^*^p < 0.05 and ^**^p < 0.01, compared with the AD group by two-way ANOVA.

**Figure 8:**
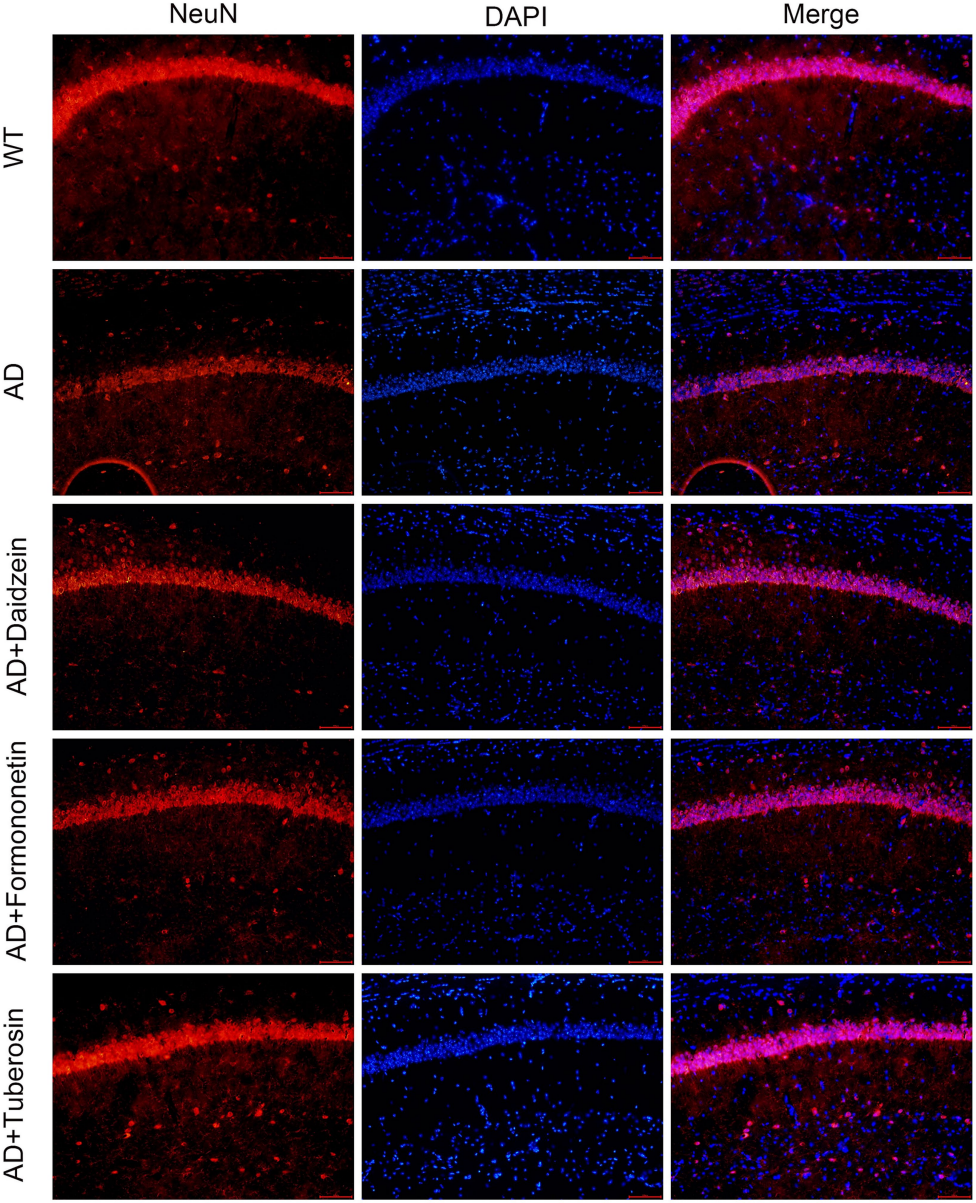
Active components of Pueraria improve neuronal loss in AD mice. Representative immunofluorescence images of NeuN, DAPI, and merge staining. The scale bar represents 100 μm.

To evaluate the role of Pueraria active components in Alzheimer’s disease (AD), we tested AD mice treated with these components using the Morris water maze (MWM), novel object recognition (NOR), and Y-maze tests (*n* = 8 for each test). In the MWM test (Figure 7A), AD mice injected with Pueraria active components (daidzein, formononetin, tuberosin) required less time to reach the platform compared to untreated AD mice. Furthermore, on the sixth day of testing, treated AD mice spent more time in the target quadrant (Figures 7B,C) and crossed the platform more frequently (Figure 7D) compared to the untreated group, suggesting improved learning and memory. In the NOR test (Figures 7E,F), AD mice treated with Pueraria active components spent more time exploring the novel object and made more visits compared to untreated AD mice, indicating improved recognition memory. In the Y-maze test (Figures 7G,H), treated AD mice also spent more time in and traveled a greater distance in the new arm, further suggesting improvements in spatial memory. Additionally, NeuN staining was used to label neurons in the hippocampal CA1 region. Compared to wild-type (WT) mice, AD mice exhibited significant neuronal loss in the hippocampal CA1 region. However, AD mice treated with Pueraria active components (daidzein, formononetin, tuberosin) showed varying degrees of increased neuronal numbers (Figure 8), indicating that the active components help mitigate neuronal loss. These results demonstrate that Pueraria active components improve learning and memory deficits and reduce neuronal loss in AD mice.

## 4 Discussion

This study employed an integrated approach combining network pharmacology, machine learning, and experimental validation to systematically elucidate the molecular mechanisms underlying the therapeutic effects of Pueraria against Alzheimer’s disease (AD). Network pharmacological analysis identified 45 overlapping targets between Pueraria and AD, representing key convergence points in the complex pathological networks of neurodegeneration. The integration of 127 distinct algorithmic combinations through machine learning established a robust framework for therapeutic target prioritization, with the RF + XGBoost model demonstrating superior performance across multiple independent validation datasets (AUC = 0.792). Notably, PFKFB3 emerged as the most promising therapeutic target, exhibiting the highest diagnostic value (AUC = 0.805) and showing significant causal relationships with AD through Mendelian randomization analysis. Experimental validation further confirmed that Pueraria’s bioactive compounds—daidzein, formononetin, and tuberosin—effectively ameliorated Abeta_1-42_-induced cognitive dysfunction and neuronal loss in AD mouse models, bridging computational predictions with biological reality.

Our findings provide strong empirical support for the core principles of traditional Chinese medicine’s “multi-component, multi-target, multi-pathway” therapeutic intervention, particularly in addressing complex neurodegenerative diseases. Functional enrichment analysis revealed that the 45 overlapping targets predominantly participate in critical neurobiological processes, including synaptic plasticity, GABA-A receptor activity, and GABAergic synaptic transmission—pathways that are severely disrupted in AD (23, 24). This systems-level approach contrasts with previous reductionist studies focusing primarily on single components, representing a shift toward a more holistic phytochemical constituent analysis.

The identification of PFKFB3 (6-phosphofructo-2-kinase/fructose-2,6-bisphosphatase 3) as a primary therapeutic target highlights the role of glucose metabolic dysfunction in AD pathogenesis. In healthy neurons, PFKFB3 maintains low expression levels through APC/C-Cdh1-mediated proteasomal degradation, inhibiting glycolysis and promoting a pentose phosphate pathway (PPP)-dominated metabolic profile that ensures NADPH generation and glutathione regeneration to maintain redox homeostasis (25, 26). During AD progression, Aβ fragments and excitotoxic stimuli inhibit APC/C-Cdh1, leading to abnormal stabilization of PFKFB3, driving metabolic reprogramming and compromising the PPP’s antioxidant capacity (27–30). This results in NADPH depletion, mitochondrial ROS burst, oxidative stress, neuronal apoptosis, and ultimately, synaptic loss and cognitive decline (31). This mechanism was validated in AD models, where transgenic mice exhibited significantly elevated PFKFB3 expression following amyloid plaque deposition, accompanied by astrocyte activation and neuronal dysfunction (32). Correspondingly, Aβ-treated cortical neurons demonstrated dose-dependent PFKFB3 accumulation and cell death (33). The small molecule inhibitor AZ67, targeting this pathway, exhibited neuroprotective effects by blocking Aβ-induced glycolytic hyperactivation, restoring NADPH levels, and eliminating mitochondrial ROS, thereby preventing neuronal apoptosis (34). Its *in vivo* advantages include efficient blood–brain barrier penetration and neuronal selectivity.

Our research suggests that Pueraria exerts therapeutic effects in AD through inhibition of PFKFB3 expression. Comprehensive behavioral assessments confirmed that Pueraria’s core bioactive constituents (daidzein, formononetin, tuberosin) significantly ameliorated cognitive dysfunction in Abeta_1-42_-induced AD mice. In spatial memory tests (Morris water maze), treated mice exhibited reduced escape latencies, increased target quadrant dwelling times, and more platform crossings, indicating restored spatial learning and memory. These findings align with network pharmacological predictions that Pueraria modulates GABAergic synaptic pathways, a fundamental mechanism for hippocampus-dependent memory. Additionally, novel object recognition (NOR) and Y-maze tests validated the enhancement of non-spatial working memory in AD mice treated with Pueraria. Immunofluorescence results further demonstrated that Pueraria treatment reduced neuronal loss in the hippocampal CA1 region. Combined with mechanistic studies, these neuroprotective effects are likely mediated through metabolic reprogramming, where inhibition of PFKFB3 reverses Aβ-induced glycolysis/PPP imbalance, reduces NADPH depletion and ROS accumulation, and maintains synaptic function via Pueraria-activated p38MAPK-CREB pathways that promote synaptin expression and synaptic plasticity (34). Moreover, neuroinflammation is likely alleviated through targeted inhibition of microglial PFKFB3, providing synergistic benefits for the neuroinflammatory microenvironment (35). The distinct yet consistently beneficial effects of daidzein, formononetin, and tuberosin suggest structure–activity relationships warranting further exploration for therapeutic optimization. These findings position Pueraria within the broader context of natural product-based AD therapeutics, offering advantages in safety profiles, multi-target engagement, and long-term use potential—important considerations given the chronic nature of neurodegenerative diseases and the limitations of current synthetic drugs.

Several important limitations must be considered when interpreting our findings. While network pharmacological predictions were robust, systematic experimental validation of the remaining 16 core targets beyond PFKFB3 is necessary to fully realize the therapeutic potential of our multi-target approach. Despite rigorous batch effect correction, the use of public transcriptomic datasets may introduce limitations related to sample heterogeneity and technical variation, which may not fully capture AD’s complexity across diverse patient populations. Additionally, while the Aβ_1-42_ injection model is well-established, it may not fully recapitulate the chronic progression observed in human AD, particularly with respect to amyloid accumulation and tau pathology. Lastly, further research into the pharmacokinetics and bioavailability of Pueraria compounds, including blood–brain barrier penetration and brain tissue distribution, is essential for optimizing therapeutic formulations and dosing strategies.

## 5 Conclusion

In summary, this study demonstrates the potential of Pueraria as a neuroprotective functional food for Alzheimer’s disease (AD). Through network pharmacology, we identified 45 overlapping targets between Pueraria and AD. Integrating machine learning and Mendelian randomization, PFKFB3 emerged as a central target. Molecular docking revealed that key bioactive compounds—daidzein, formononetin, and tuberin—exhibited strong binding affinities to PFKFB3. *In vivo* experiments further confirmed that Pueraria administration enhanced cognitive performance and reduced neuronal loss in AD model mice. Collectively, these findings support Pueraria as a promising dietary intervention that could complement and potentially enhance current therapeutic strategies for AD.

## Data Availability

Publicly available datasets were analyzed in this study. This data can be found here: https://www.ncbi.nlm.nih.gov/geo/query/acc.cgi?acc=GSE5281, https://opengwas.io/datasets/ieu-b-5067, http://herb.ac.cn/Detail/?v=HERB001874&label=Herb, and https://www.swisstargetprediction.ch/result.php?job=640981954&organism=Homo_sapiens.
